# Correction: Enhanced performance of porous forward osmosis (FO) membrane in the treatment of oily wastewater containing HPAM by the incorporation of palygorskite

**DOI:** 10.1039/d1ra90177j

**Published:** 2021-12-24

**Authors:** Qianwen Zhang, Wande Ding, Huanzhen Zhang, Kefeng Zhang, Zhili Wang, Jiayu Liu

**Affiliations:** School of Water Resources & Environment, China University of Geosciences Beijing 100083 China dingwande18@sdjzu.edu.cn; School of Municipal and Environmental Engineering, Shandong Jianzhu University Jinan 250101 China; Shandong Shuifa Environmental Technology Co., Ltd Jining 272000 China; Center of Environment Engineering Consulting, Chinese Academy of Environmental Planning Beijing 100041 China

## Abstract

Correction for ‘Enhanced performance of porous forward osmosis (FO) membrane in the treatment of oily wastewater containing HPAM by the incorporation of palygorskite’ by Qianwen Zhang *et al.*, *RSC Adv.*, 2021, **11**, 22439–22449, DOI: 10.1039/D1RA02858H.

The authors regret that the affiliations for co-author Qianwen Zhang were incorrectly given in the original article. The correct affiliations are shown here.

The Royal Society of Chemistry apologises for these errors and any consequent inconvenience to authors and readers.

## Supplementary Material

